# Insight into the Possible Use of the Predator *Bdellovibrio bacteriovorus* as a Probiotic

**DOI:** 10.3390/nu12082252

**Published:** 2020-07-28

**Authors:** Giulia Bonfiglio, Bruna Neroni, Giulia Radocchia, Massimiliano Marazzato, Fabrizio Pantanella, Serena Schippa

**Affiliations:** Public Health and Infectious Diseases Department, Microbiology section, “Sapienza” University of Rome, Piazzale Aldo Moro, 5, 00185 Roma, Italy; Giulia.bonfiglio@uniroma1.it (G.B.); bruna.neroni@uniroma1.it (B.N.); Giulia.radocchia@uniroma1.it (G.R.); massimiliano.marazzato@uniroma1.it (M.M.); fabrizio.pantanella@uniroma1.it (F.P.)

**Keywords:** predator, probiotic, ecological equilibrator, microbiota, dysbiosis

## Abstract

The gut microbiota is a complex microbial ecosystem that coexists with the human organism in the intestinal tract. The members of this ecosystem live together in a balance between them and the host, contributing to its healthy state. Stress, aging, and antibiotic therapies are the principal factors affecting the gut microbiota composition, breaking the mutualistic relationship among microbes and resulting in the overgrowth of potential pathogens. This condition, called dysbiosis, has been linked to several chronic pathologies. In this review, we propose the use of the predator *Bdellovibrio bacteriovorus* as a possible probiotic to prevent or counteract dysbiotic outcomes and look at the findings of previous research.

## 1. Introduction

The gut, the largest interface (200 m^2^) between the host and the environment, is devoted to the metabolism of nutrients and water absorption and is colonized by trillions of bacteria, archaea, fungi [1], and viruses [2,3] that coexist in a complex ecosystem called the gut microbiota [4]. This microbial community equips the host with a pool of bacterial genomes, called the microbiome, with a coding capacity 150-fold higher than that of the human genome, providing functional features that humans have not evolved with [5]. The human genome and microbiome merge to form the hologenome, which determines the metabolic characteristics of the organism [6]. The intestinal microbial ecosystem is nowadays considered to be a real organ that contributes to nutrient metabolism, stimulates the immune system, and helps the host in its defense against pathogens in order to maintain community integrity [7,8]. The principal phyla composing a healthy adult gut microbiota have been determined: Firmicutes and Bacteroidetes make up 70% of the total microbiota, with Proteobacteria, Actinobacteria, Verrucomicrobia, Fusobacteria, and Cyanobacteria found in decreasing percentages, respectively [9,10]. At the taxonomic level of species, the gut microbiota composition varies between individuals so much that it is comparable to a fingerprint [11,12]. The metabolic functions of the gut microbiota include the degradation of complex polysaccharides and the synthesis of short-chain fatty acids (SCFAs), amino acids, and vitamins that allow the human host to utilize many energetic sources. SCFAs produced by commensal bacteria seem to regulate, via epigenetic mechanisms, the immune equilibrium between the host defense system and the maintenance of commensal immune tolerance [13]. The microbiota also contributes to the maintenance of the intestinal epithelium-barrier integrity by regulating enterocyte turnover, maintaining cell-to-cell junctions, and promoting epithelial repair following injury [14]. Once established, the composition of the gut microbiota should ideally remain stable throughout adult life. However, it is continuously influenced by many external factors, such as stress [15], aging [16], lifestyle and diet [17], use of antibiotics [18], and health status [19]. These factors harm the normal intestinal microflora, resulting in alterations to bacterial metabolism, as well as overgrowth of potentially pathogenic microorganisms and deleterious changes in their local distribution [20]. Nobel prize laureate Elie Metchnikoff (1845–1916) defined the condition in which “good” bacteria are no longer able to control “bad” bacteria as dysbiosis, whereas the presence of a balanced gut microbiota is known as a state of eubiosis [21]. The growth of potentially pathogenic bacteria in the intestine can provoke the release of toxic products that contribute to chronic and degenerative diseases [20]. A common feature of dysbiosis is the loss of microbiota diversity (LOMD) [4] that has been correlated with several pathologies like inflammatory bowel disease, celiac disease, obesity, metabolic disorders, and new associations that continue to be discovered [12]. Following the recognition of the role of a balanced gut microbiota in maintaining a healthy status, several strategic therapies have been developed that aim to restore or maintain the eubiotic state of the intestinal microbial ecosystem. Therapeutic strategies currently adopted include the use of probiotics or administration of prebiotics (formulations of substrates metabolized by probiotic bacteria with the aim of favoring their growth) or the use of their combinations (called symbiotics) [5]. Recently, other original therapeutic approaches have been proposed, like fecal microbiota transplantation (FMT) [22,23], phage therapy, and the use of predatory bacteria. Phages are bacteria-infecting viruses that can influence bacterial populations in microbial communities. Phages represent almost 90% of the human virome and have been proposed as an alternative tool to antibiotics therapies to modulate the composition of microbial ecosystems [22]. The idea of using predatory bacteria has the same rationale as phage therapy: exploiting what normally happens in nature to counteract bacterial overgrowth in ecosystems. Bacterial communities control and shape bacterial populations in a wide range of natural and man-made environments [23,24]. Bacterial predation has been proposed to be one of the selective forces that plays an important role in maintaining the biodiversity of ecosystems [25,26,27,28,29,30]. This review will provide insight into the predator *Bdellovibrio bacteriovorus* with the purpose of presenting the current knowledge about its potential clinical use as a probiotic or therapeutic agent to control bacterial populations within the gut. 

## 2. Discovery of Predators and Characteristics

*Bdellovibrio bacteriovorus* is an aerobic Gram-negative bacterium that is a predator of other Gram-negative strains. It inhabits terrestrial, marine, and biotic environments where, through its predatory activity, it preserves the stability of the microbial ecosystem, playing the role of an ecological balancer [31,32,33]. However, recently, it has been shown that under microaerophilic conditions, it seems to be able to survive and maintain its predatory activity, and, in anaerobic conditions, it can still survive, but it loses its ability to act as a predator [34,35].

*B. bacteriovorus* was described, for the first time, by Stolp and Starr as an ectoparasite capable of attacking the outer membrane of susceptible bacterial prey, causing lysis of the cell [36]. It was found during an attempt to isolate bacteriophages from soil by Stolp and Petzold, who noted late-developing plaques that increased in size during prolonged incubation and appeared as small, comma shaped, highly motile bacteria under microscope observation [37].

Since then, predators have been isolated from many different ecosystems in widely dispersed geographical areas, such as salt water and fresh water, the plant rhizosphere, soil, sewage, and animal guts, leading to the conclusion that these predatory bacteria have an ubiquitous distribution [34,35,36,37,38,39,40,41,42]. Ideally, Bdellovibrio strains could be found in every site where Gram-negative bacteria, representing their food supply, are present [35,37,38,40,43,44,45,46,47,48]. Predators seem to play an essential role as ecological balancers in the environment, shaping the microbial community [49,50,51,52,53]. As the knowledge of these microorganisms has increased, the diversity among isolates has required the classification of some groups into distinct species and genera. All isolates were named Bdellovibrio-like organisms (BALOs) and are now classified based on genomic and phenotypic criteria, as follows: (i) the 16S rRNA gene sequence [54], (ii) the β-subunit of the bacterial RNA polymerase (*rpoB*) gene sequence [48], (iii) the GC content (%), and (iv) the quantity of sodium chloride required for growth [55]. BALOs are present mainly in two classes: Alphaproteobacteria, which includes the genus *Micavibrio*, and Deltaproteobacteria, containing five families: Bdellovibrionaceae (including *Bdellovibrio bacteriovorus* HD100 and *Bdellovibrio exovorus JSS*), Peredibacteraceae (including *Peredibacter starrii*), Bacteriovoracaceae (including *Bacterivores stolpii*)*,* Pseudobacteriovoracaceae (with the recent isolate *Pseudobacteriovorax antillogorgiicola* [56]), and Halobacteriovoraceae (including *Halobacteriovorax marinus SJ* and *Halobacteriovorax litoralis JS5* [48,54,56,57,58,59,60,61]). BALOs can also be epibiotic predators, such as *Bdellovibrio exovorus* and *Micavibrio aeruginosavorus* [62,63], which attach to the outer membrane of the prey’s cell, take all the nutrients, and divide in a binary manner, remaining in the extracellular environment. Alternatively, they can be periplasmic predators, such as *B. bacteriovorus*, that invade the outer membrane and insert themselves into the periplasmic space from which they feed on prey and reproduce, causing prey lysis during release of the progeny [64].

### 2.1. The Predatory Life Cycle of Bdellovibrio Bacteriovorus

*Bdellovibrio bacteriovorus* is an obligate predator with a biphasic and a dimorphic cell cycle: (i) a free-living form, characterized by the presence of a polar flagellum, named the attack phase (AP) and (ii) a form without a flagellum, within the prey, named the growth phase (GP). Briefly, once the prey is encountered, the predator invades the periplasmic space of Gram-negative bacteria by attacking the prey’s external surface and hydrolyzing its components, forming the bdelloplast [34].The prey’s periplasmic space represents the *B. bacteriovorus* replicative niche. The progeny of flagellated bacteria can lyse the host cell and start a new cycle of predation. The prey’s cell wall [58,59,61,62,63] and predator’s flagellum [63,64] undergo numerous modifications in the transition between the first and the second phase. Once inside the periplasmic space, the predator grows as a single multinucleoid filament that will split into the progeny [65,66,67]. The predatory mechanism seems to depend on two distinct prey-related signals: the triggering of the beginning of the process and the AP-to-GP transition. The signal for the start of the process is still unknown but seems to be related to the penetration of the prey, bdelloplast formation, and the prey’s nutrient consumption [67]. The second signal, released by the prey, is hypothesized to be soluble and to promote DNA synthesis, which is essential for the sustainment of the actively growing phase [68,69]. Rotem and collaborators revealed the existence of an intermediate phase between the first and the second signal that seems to be decisive for the start of the GP phase by the predator. Furthermore, authors proposed a molecular model of the signal cascade that brings about the predatory process [70]. According to the model, in the early AP, free-living and fast-swimming Bdellovibrio cells up-regulate the *bd0108* gene involved in the initiation of the predation process and in the regulation of type IVa pili extraction/retraction. This gene is indispensable for predation and also prey entry [71]. A highly expressed putative cyclic di-GMP riboswitch (*mer*RNA) and *fli*C(1–5) genes responsible for predator movement were also found to be upregulated in early AP cells [72]. When prey and predators meet, AP predatory cells prepare to switch to GP cells, reducing *mer*RNA and *bd*0108 expression, probably in response to a transduction signal. Meanwhile, there is an increase in the expression of genes, such as *bd0816* and *bd3459*, encoding enzymes able to generate a pore in the outer membrane of the prey [69,73]. Other genes (*pcnB, dgcB, rpoD*, and *rpoH*) are induced in order to prepare the predator cells for the GP [70]. To complete the transition to the GP, the second soluble signal from the cytosol prey is required. Bdelloplast’s cytoplasmic membrane becomes permeable to small molecules [70,74], including prey-derived nutrients [75,76]. When both signals are sensed by the predator, the bdelloplast formation is concluded, and the AP-related genes are shut down, while GP-related genes are up-regulated [74,76]. Karunker’s group demonstrated an increase in active transcription from AP to GP (33% versus 85%) [77]. The whole process lasts 10–20 min. It is also assumed that prey would have difficulty becoming resistant to predation [78].

### 2.2. Prey Range

BALOs are small, Gram-negative predatory bacteria that prey on other Gram-negative species. Some have a broad-range spectrum; others prey on only a few different species [47,48,79,80]. Several studies have aimed to discover their prey’s host range. In one of the first studies that aimed to establish the susceptibility of various species to the predators, Taylor and collaborators tested the predation preferences of 13 marine BALO isolates recovered from coast of Oahu, Hawaii against 42 marine and terrestrial bacterial strains. Many of the bacterial species were found to be susceptible to predation by all of the BALO isolates. The *Vibrio* species was found to be the most susceptible, while the *Pseudomonas* species was found to be resistant [47]. Using plant pathogenic and plant growth-enhancing bacteria as prey, Jurkevitch and collaborators showed that soil and rhizosphere *Bdellovibrio* isolates have different utilization patterns, even for related prey [81]. *Bdellovibrio bacteriovorus* was reported to prey on strains belonging to the *Escherichia, Pseudomonas, Chromatium*, and *Spirillum* genera [81]. More recently *B. bacteriovorus* 109J was found to be able to attack species from the genera *Acinetobacter, Aeromonas, Bordetella, Burkholderia, Citrobacter, Enterobacter, Escherichia, Klebsiella, Listonella, Morganella, Proteus, Pseudomonas, Salmonella, Serratia, Shigella, Vibrio*, and *Yersinia* [82]. BALOs prey on Gram-negative bacteria, but *B. bacteriovorus* does not prey on itself, even if grown axenically as prey and cocultured with a predator strain, meaning that its outer membrane differs from that of other Gram-negative bacteria [83]. Schwudke et al. found that the lipid A in *B. bacteriovorus* presents a substitution of a mannose for phosphates, which leads to the loss of a negative charge in the typically polar head group, allowing close access to the outer membrane of prey [84]. This could be the modification that marks Bdellovibrio as non-prey and explains its lower susceptibility to cationic antibiotics and antimicrobial peptides. It also has a higher sensitivity to Triton X-100 than other Gram-negative bacteria [78,85]. Finally, it has been recently reported that *B. bacteriovorus* interacts with Gram-positive bacteria, such as *S. aureus* [86,87].

## 3. Mathematical Model of Predation

The current working model for community dynamics is the Lotka–Volterra model, which predicts that predators will rapidly and significantly reduce the populations of the most abundant species, preventing the overgrowth of species among others and guaranteeing species diversity in the ecosystem. Combining the Lotka–Volterra model of population dynamics with regression techniques offers a mechanistic scheme that is useful for constructing predictive models of real ecosystem dynamics [4]. A mathematical model describing *B. bacteriovorus* that best fits experimental data and seems to realistically predict the predation cycle length was developed by Hobley [88]. The model considers bdelloplasts as a separate and transient population and shows that a significant proportion of bdelloplasts do not lyse and release viable progeny, in contrast to what was previously assumed [89,90,91]. Although Hobley’s model is based on an idealized environment, it is the one that is most practical for using *B. bacteriovorus* as an antimicrobial agent. Another study examined the behavior of *B. bacteriovorus* against *Burkholderia stabilis* in a structured environment consisting of sand under various regimes of wetness and showed that predation dynamics are influenced by environmental conditions [92]. This is relevant considering that in vivo environments are more structurally complex than buffer systems. Baker and collaborators showed that there are differences between predation in buffer and serum, highlighting both the potential and limitations of *B. bacteriovorus* HD100 acting therapeutically against *K. pneumoniae* in serum, stimulating future research into the medicinal behaviors and dosing of this living antibacterial [93]. A comprehensive mathematical framework should consider interactions with the immune system, environmental conditions, and interactions with commensals in order to provide guidance for in vivo application and help researchers to design further dose schedules and therapeutic tests of predators. For this purpose, modelling should be firmly based on experimentally quantified results of predation in the host; this requires further research.

### Prey Resistance and Predator Escape

A predator’s inability to prey on a specific host can be due to its structural features, which inhibit attachment, penetration, or replication, or due to the escape strategy of the host. Dashiff and collaborators demonstrated that the prey’s host can also modify its surrounding environment, making it less suitable for predator survival and proliferation. In particular, the metabolism of specific carbohydrates by prey can acidify the environment to a level that is toxic to the predator, disabling predation [94]. *B. bacteriovorus* can never entirely get rid of its prey [90,95]. Shemesh and Jurkevitch showed that a transiently resistant population persists and, after predator removal, reverts to a sensitive phenotype. These authors supposed that the cause of this plastic resistance is due to a physiological need rather than a mutational event [53]. Moreover, the chance for the prey to acquire resistance factors has been examined. Lambert and collaborators showed that *B. bacteriovorus* possesses a self-protection protein that inhibits its endopeptidase, which is used to digest the prey’s cell wall during membrane transit, but they also revealed that even when the predator’s self-protective gene is transferred to the prey cell, it is not protected against the predator [96]. Additional structures exterior to the outer membrane can prevent predation inhibiting *B. bacteriovorus* attachment [78]. Although the bacterial capsule represents a virulence factor for the owner strain, it does not seem to affect *B. bacteriovorus* 109J predation, as shown by Koval and Bayer [97]. These authors proposed that predators can penetrate the *E. coli* K29 glycocalyx capsule through the motile force generated by flagellar movement. Other experiments should be done to assess the veracity of this assertion for other capsulated prey. Another possible barrier for *B. bacteriovorus* attachment could be the S-layer, a cell envelope-associated structure composed of monomolecular arrays of protein or glycoprotein subunits expressed by many types of bacteria [98,99]. The S-layers of *Aquaspirillum serpens*, *Aquaspirillum sinuosum*, and *Aeromonas salmonicida* were found to be protective against *B. bacteriovorus* 109J predation, although the authors of that study hypothesized that prolonged subculture of the prey could cause the exposure of some areas of the outer membrane that can be used as attachment sites by predators [100]. Another virulence factor that could be used by prey as a strategy to avoid predation is the ability to produce biofilm, surface-associated communities of bacterial cells with an extracellular polymeric matrix, even known to be responsible for antimicrobial resistance. Several studies have demonstrated that both single-species and multiple-species biofilms are susceptible to *B. bacteriovorus* attack [33,86,87,94,101]. The ability of a predator to penetrate biofilms deeply and destroy them is the characteristic that distinguishes it from other biological antibacterial tools [102]. *B. bacteriovorus* 109J was found to be able to damage metabolically inactive biofilms and eradicate biofilms developed on hydroxyapatite surfaces [103]. Kadouri’s group also reported that *Bdellovibrio* is more efficient in preying biofilms than planktonic cells and that the efficiency of predation is directly proportionated to the prey density [82]. All of these findings strongly support the idea of using *B. bacteriovorus* as a biological army for the treatment of disease-related biofilm and infections.

## 4. Potential Therapeutic Use

The discovery, commercialization, and administration of antibiotics to treat infections revolutionized modern medicine, but, at the same time, its indiscriminate usage and easy availability led to increased antimicrobial resistance among common bacterial pathogens, so much so that it is now threatening its therapeutic achievement, risking the successful outcomes of critically ill patients [104,105]. The World Health Organization declared antibiotic resistance as one of the three most important public health threats of the 21st century [106]. In addition to the Gram-positive *Staphylococcus aureus,* many of the multidrug-resistant infections are caused by pan-resistant Gram-negative bacterial strains, which are currently considered untreatable with conventional antibiotics. These are named ESKAPE pathogens and they include Enterobacter, *Klebsiella pneumoniae*, *Acinetobacter baumannii*, *Pseudomonas aeruginosa*, and *Escherichia coli* [107,108,109]. In response to the emergent increase of antimicrobial-resistant bacterial infections as a global health issue, numerous options are being examined to treat drug-resistant bacterial infections. Among these, recently, the use of *Bdellovibrio bacteriovorus* has been taken into consideration [110]. Sun and collaborators examined the ability of *B. bacteriovorus* to prey on multidrug-resistant (MDR) and extensively drug-resistant (XDR) Gram-negative clinical bacteria like *Klebsiella pneumoniae, Escherichia coli, Acinetobacter baumanni*, and *Pseudomonas aeruginosa* in sessile and planktonic culture. *B. bacteriovorus* 109J was found to be able to prey on all planktonic cultures tested, with the most efficient predation observed for drug-resistant *E. coli*, which demonstrated a promising efficacy in preventing biofilm formation [111]. Recently, it was demonstrated that *B. bacteriovorus* is also able to prey on the *Burkholderia cepacia* complex, suggesting its utility in the biocontrol of both human and plant pathogens [112]. The application of predators on periodontal infection, usually caused by Gram-negative pathogens, has also been shown. Van Essche and collaborators evaluated the ability of six different BALOs to prey on six periodontopathogens (*Aggregatibacter actinomycetemcomitans, Eikenella corrodens, Porphyromonas gingivalis, Capnocytophaga sputigena, Prevotella intermedia,* and *Fusobacterium nucleatum*), concluding that the oral application of a high concentration of BALO may rapidly decrease a wide range of periodontal pathogens from the mixed oral microbiota. In addition, they found that predatory therapy should be considered for the development of an adjuvant to standard periodontal therapy, although other investigations are still needed [113]. Moreover, there is also the fact that the commensal periodontal flora is mainly composed of Gram-positive species, which are supposed to be resistant to predation. The ability of *B. bacteriovorus* to prey on the *P. aeruginosa* multidrug-resistant strain isolated from respiratory samples of cystic fibrosis patients was recently shown [86], suggesting that *B. bacteriovorus* could be aerosolized and used to counteract Gram-negative infections in cystic fibrosis patients. Having been demonstrated to attack members belonging to the genus *Proteus*, it could even be useful for the treatment of urinary-tract infections, especially in catheterized patients [94,114]. It seems also to have an inhibitory effect against the biofilm formation of *Staphylococcus aureus* and, through some still unknown manner, against its growth [86,87,115]. Our research group recently showed the ability of *B. bacteriovorus* HD100 to attack the Adherent Invasive *Escherichia coli* (AIEC) strain LF82, in both planktonic and sessile forms, pointing out its ability to interfere with some points of LF82 pathogenic dynamics, for example its ability to adhere to and invade intestinal cells. This provides a basis for its possible use as a therapeutic approach to control/eliminate AIEC strains from Crohn’s disease patients [33].

### 4.1. In Vitro Interaction with Immune System and Host Tissues

The first consideration for the possible use of *B. bacteriovorus* as a probiotic is how the immune system would respond to the administration of this bacterial strain. Schwudke and collaborators showed that the exclusive lipopolysaccharide (LPS) of *B. bacteriovorus* HD100 does not seem to induce a strong inflammatory response, probably due to the absence of negatively charged groups in its lipid A, leading to a low bond affinity for LPS receptors on human cells [84]. These authors also shown that human mononuclear cells are stimulated by *B. bacteriovorus* to produce lower tumor necrosis factor alpha (TNF-α) levels with respect to *E. coli* K12 stimulation [84]. With the purpose of shedding light on interactions between predatory bacteria and human cells, Monnappa and collaborators investigated the potential inflammatory or cytotoxic effects following the contact of predators with several different mammalian cell lines. Three human intestinal cell lines (HT29, Caco2, and T84 cells), the lung epithelial cell line NuLi-1, and the RAW 264.7 murine cell line were exposed to *B. bacteriovorus* HD100 for 24 h. The results showed a minimal reduction in cell viability on epithelial cell lines, whereas murine macrophages were not impacted. The results also showed that *B. bacteriovorus* stimulates the production of proinflammatory cytokines, such as TNF-α and interleukin-12 (IL-12), but at a lower magnitude compared to *E. coli* [116]. In the same year, Gupta and collaborators challenged other human cell lines, such as adherent human keratinocyte cell line (HaCaT), human liver epithelial cells (HepG2), human kidney epithelial cells (HK-2), loosely adherent human spleen monocytes (MD), and a suspension of human blood monocytes (THP-1), with high doses of the predator and measured any changes in cell viability and the inflammatory response [117]. For all cell lines tested, cell viability when exposed to the predators was significantly higher than that obtained when exposed to the *P. aeruginosa* positive control. *B. bacteriovorus* did not cause any significant alterations in the cytokine levels for the four cell lines tested, but it elicited cytokine production in activated blood macrophages, although the induction of IL-1β and TNF-α was lower than with *E. coli* exposed cells [117]. Raghunathan and collaborators studied the interactions of live *B. bacteriovorus* HD100 with human phagocytic cells (PMA-differentiated U937 cells) to determine the uptake mechanisms, its persistence inside phagocytes, and associated cytokine responses [110]. U937 cells were found to passively engulf *B. bacteriovorus*, which persisted within for 24 h without affecting cell viability. Eventually, they were trafficked through the phagolysosomal pathway of degradation. This study provides new knowledge on predator interaction with the immune system and its availability inside the host [110].

### 4.2. In Vivo Interaction with the Host

Progress toward the use of *B. bacteriovorus* in vivo, as a potential living antibiotic, requires research on predators in biologically relevant systems, including investigations of their persistence and effects on the host, possible changes to native microflora, and study of the dynamics of predation at the site of infection. The first report of in vivo inoculation of *Bdellovibrio bacteriovorus* with therapeutic purpose dates back to the 1970s when Nakamura’s group reported the ability of this species to reduce the severity of keratoconjunctivitis induced by *Shigella flexneri* in rabbit eyes, if injected within 48 h of *Shigella* infection. Coadministration of the predator and prey in ligated ileal loops of rabbits resulted in attenuated *S. flexneri* enterosorption, probably as a result of a reduced load due to predation [118]. Predatory bacteria administered on the ocular surface of rabbit eyes had no toxic effects in either the presence or absence of corneal epithelial abrasions and did not preclude corneal epithelial wound healing or increase clinical inflammatory signs in vivo [119]. Shatzkes and collaborators determined the safety of respiratory and intravenous inoculations of *B. bacteriovorus* 109J in mice, finding no reduction in mice viability. They also evaluated the cytokine response by RT-qPCR and ELISA and found a modest inflammatory response at 1  h post injection in the respiratory tract of mice, but this was not sustained at 24  h. Intravenous injection caused an increase in proinflammatory cytokines at 2 h post injection, but the levels returned to baseline by 18 h [120]. No morbidity or adverse histopathology of various organs were observed following intravenous inoculations of predators in rats, confirming their safety in this animal model. The authors reported the inability of *B. bacteriovorus* to reduce the burden of *K. pneumoniae* in the blood or prevent its dissemination to other organs by injection into the tail veins of rats at 18 or 24 h after *K. pneumoniae* infection, suggesting that predators could be ineffective for the treatment of acute blood infections [121]. The safety profile for *B. bacteriovorus* has encouraged therapeutic trials in a range of animal models. Shanks’ group found no negative effect in the *Galleria mellonella* model after injection of a high concentration of predatory bacteria into the hemocoel [122]. In our previous work, we used this model to assess the safety of *B. bacteriovorus* HD100 and its possible protective effect against Adherent Invasive *E. coli* (AIEC) infection. Our results suggested that *B. bacteriovorus* is not toxic to the larvae, and pretreatment of *G. mellonella* with the predator seemed to be protective against AIEC infection, opening hope for its future therapeutic use [33]. Willis and collaborators injected *B. bacteriovorus* HD100 into zebrafish larvae infected with lethal doses of *Shigella flexneri* and noticed an increase in zebrafish survival. The study indicated that the predator was efficiently engulfed and eliminated by host immune cells. However, predator activity significantly contributes to zebrafish survival [123]. Recently, Findlay and collaborators demonstrated the ability of *B. bacteriovorus* HD100 to prey on *Y. pestis* in vivo and protect mice from lethal plague. Using whole body imaging, the authors also noticed an adipose tissue tropism of *Bdellovibrio* in vivo that had never been reported before. Instead, similar to what was previously reported, human phagocytic cells produced lower proinflammatory cytokine levels when infected with *B. bacteriovorus* compared to member strains of Enterobacteriaceae family [124]. All these studies support the idea of using this predator as a therapeutic agent to counteract Gram-negative infections.

## 5. Predators in the Gut

In the gastrointestinal (GI) tract, the microbiota is not distributed homogeneously, and it varies according to the considered site. The different characteristics of the GI tract influence microbial colonization [125]. Anaerobic bacteria are predominant in the guts of healthy subjects [126]. The intricate networks among microbes, living in the intestine of mammals where there is a specific oxygen gradient related to the specific anatomical portion, could create microenvironments that are favorable to both aerobic and anaerobic growth [127]. For example, facultative anaerobic bacteria and even aerobic bacteria by consuming oxygen, generate anaerobic niches. Patini et al. showed that the predatory capacity of *Bdellovibrio* is compromised by conditions of anaerobiosis [34], although it can survive under anoxic conditions and is able to grow and attack under microaerophilic conditions [34,35]. To date, the presence of aerobic bacteria has not been clearly demonstrated, but, in this complex network where we still don’t know the complicated relationships between microbes and the environment around them, we cannot exclude the presence of niches with oxygen, necessary for the growth of aerobic bacteria. In pathological states, a dysbiotic condition can occur, characterized by the loss of mutualistic relationships among microorganisms. The majority of dysbiotic states are characterized by the overgrowth of aerobic and anaerobic bacteria, including potentially pathogenic germs [128]. This indicates that when dysbiosis conditions occur in intestinal habitat, aerobic growth is encouraged. In 1977, Westergaard and Kramer published the first investigation of the fate of Bdellovibrio cells (strain MS7, isolated from wastewater) administered to fish, frogs, and mice. Bdellovibrio cells were recovered in small quantities only after 6 days from the inoculation of predators into the esophagi or stomachs of fishes. Recovery from force-fed frogs was higher in the first 15 min after administration, whereas predator survival diminished significantly within 48 h. From mice forced to drink Bdellovibrio-containing water, the authors did not recover any predator cells, meaning that the Bdellovibrio strain MS7 cannot establish itself as a member of commensals, and it could be a transient resident of the intestinal microflora of mice [129]. Early studies of Bdellovibrio associations with mammals were not promising [38] as they showed that bdellovibrios were aerobic obbligate and did not survive passage through an animal’s gut. However, a recent study [3] showed that bdellovibrios can be isolated from feces, indicating that they are present in the guts of humans and other mammals and leading us to suppose that earlier studies were simply using strains that did not colonize humans [114]. *Bdellovibrio bacteriovorus* has been found in the intestines of cows, horses, pigs, and ducks and seems to be constantly excreted through feces into the environment [130]. Iebba and collaborators’ study assessed the presence of *B. bacteriovorus* in the human gut microbiota and evaluated bioptic samples collected from Inflammatory Bowel Disease (IBD) and Celiac disease patients, and fecal samples from cystic fibrosis patients, then compared results obtained with the same kinds of samples from healthy subjects. *q*PCR methods were used to detect *B. bacteriovorus* at the mucosae level in healthy individuals. Furthermore, the relative abundance of the predator was found to be district-dependent. It was highest in the duodenum and gradually decreased in the caudal sense [39]. The reduced mucosal colonization of *B. bacteriovorus* in both IBD and Celiac patients could be the reason for the higher colonization at the mucosae level described in these pathologies [46]. More recently, Shatzkes’ group evaluated the effect of intrarectal inoculation of predators on rats’ gut-immune response and commensal microbiota. No adverse effects in gut histology and no increase in inflammation protein production were reported at 48 h post inoculation. 

The operational taxonomic unit (OTU), an operational definition used to classify closely related groups, corresponding to *Coprococcus eutactus*, a Gram-positive species previously found to be decreased in IBD patients [123], was the only OTU that significantly increased in rats inoculated with the predator, supporting the hypothesis of its possible therapeutic use in chronic intestinal pathologies. The authors also suggested that since *B. bacteriovorus* is an obligate Gram-negative predator, its inoculation could cause a shift in the abundance of Gram-positive and Gram-negative bacteria within the gut microbiota composition. However, no significant or remarkable changes in the Gram-positive/negative ratio linkable to predator treatment were observed. The limited population shifts in the microbiota caused by predatory inoculation in rats seem to be more favorable than the effects of antibiotics, and they seem to be mostly associated with healthy benefits, supporting the need for further research on the development of *B. bacteriovorus* as an antimicrobial agent [124]. A recent meta-analysis study evaluated the possibility of considering BALOs as indicators of a healthy microbiome in different animal host groups and environments. Their presence was associated with a significantly higher Simpson and Shannon diversity index value, and microbiome richness was correlated with BALOs abundance and richness in a set of sponges tested and in *H. vulgaris*, whereas an analysis of beta-diversity did not show a robust impact of BALOs on the microbiome community structure in any species considered. These results indicate that BALOs are potential drivers of microbiome alpha diversity by preying on species that are temporarily abundant in the microbial ecosystem [26]. Several recent studies indicating that Bdellovibrios can be present in feces and bioptic samples of humans and other mammals [46,131]. This led us to suppose that *B. bacteriovorus* could be a component of gut ecosystem, however more studies are certainly necessary to demonstrate that the gut provide adequate conditions for its persistence. Dwidar et al. defined the dual nature of *B. bacteriovorus* as “amphibiotic“ to describe its ability to undergo both probiotic and antibiotic activity [102]. The first attempt to use *Bdellovibrio bacteriovorus* HD100 as a therapeutic agent against in vivo infections was done by Atterbury’s group. They chose the well-studied poultry model used in Salmonella infection and intestinal/cecal colonization experiments to determine any therapeutic effects of Bdellovibrio treatment. Administration of the predator to 2-day-old (Salmonella-free) chicks did not cause any negative effects on birds’ health. When orally administered after *Salmonella enteritidis* infection, *B. bacteriovorus* seemed to be responsible for the reduction (1 log) in the numbers of Salmonella cells in chicken cecal contents compared with untreated controls and of a significant decrease of inflammation, produced by the infection. Although the authors suggested that multiple rounds of Bdellovibrio replication on the Salmonella prey in the gut would be required to eradicate the Salmonella infection, rather than simply reducing its load. This study demonstrated that Bdellovibrio species can survive gut conditions, at least for long enough to have a beneficial therapeutic effect [132]. In Table 1, we report the in vivo application of Bdellovibrio species.

## 6. Challenges That Should Be Considered for the Application of BALOs as Therapeutic Agents/Probiotics in the Gut

In order to be administered as probiotics, Bdellovibrios and other BALOs should be able to survive the passage through the gastrointestinal tract [133]. Among the elements that could be considered as negative factors for probiotic’s viability should be taken into account the level of stomach acidity, the degree of exposure and the concentration of bile salts, and the presence of pancreatic juices and antimicrobial peptides [133]. Further studies are necessary, although the discovery of *B. bacteriovorus* in the intestines of many animals, including humans, has led us to suppose that Bdellovibrios and other BALOs could survive in the intestinal environment. A limitation on the use of BALOs as therapeutic agents was shown in the study of Im et al. [134]. These authors observed that the *B. bacteriovorus* strain HD100 is unable to control bacteremic infections due to inactivation of its predatory activity by serum albumin and its serum osmolality, despite no alteration in its viability. However, several studies conducted in animal models showed that *B. bacteriovorus* can prey on human pathogens in sites different from the serum district (Table 1) [113,114,115,116,117,118,119]. Even if accurate investigations are still necessary, to date, the research conducted indicates that BALOs could represent good candidates as therapeutic agents/probiotics in the gut.

## 7. Conclusions

Predation is a common interaction between living organisms in all ecosystems [135] and is considered an important force, along with competition, that drives population and community ecology [136]. It has key impacts on population dynamics and community structure [137]. It has been proposed to be the motor that promotes the development and evolution of forms of life that are different from microbials by the incorporation of a small prokaryotic cell inside the eukaryotic ancestor as precursors of organelles [138,139]. Nowadays, the scientific community accepts and supports the hypothesis of bacterial predation as a selective force that drives the origin of eukaryotic cells and multicellularity, as well as the evolution of human pathogens [139,140,141,142]. Although predation is a strategy that occurred among bacteria long before the arrival of animals on the earth, animals have provided new habitats for bacteria, making the interaction between hosts and microorganisms more intricate. The 40–240 μm thick intestinal mucus layer [143] represents an excellent “hunting ground” for *B. bacteriovorus* cells travelling at a speed of 160 μm/s (in some cases even greater than 400 μm/s), according to the viscosity of the medium [144], in search of their prey [145]. The key role of *B. bacteriovorus* in the intestinal microbial community is manifested through its predatory activity in terrestrial and aquatic bacterial ecosystems [39,41,146]. The hypothesis is that in healthy subjects, this predatory activity helps to keep the colonization of the intestinal mucosa low, playing a very important role in maintaining homeostasis [46]. Probiotics are defined as “live microorganisms that, when administered in adequate amounts, confer a health benefit to the host” [120]. They are used to avoid dysbiotic outcomes in the presence of predisposing conditions, such as stress, chronic diseases, or prolonged use of antibiotic therapies by returning the gut to a eubiotic state [5]. Considering bacterial predation as an alternative strategy to the restoration of a eubiotic state, different studies have proposed the use of the predator *B. bacteriovorus* as a possible probiotic [33,102,114]. Assessment of safety, functionality, and stability are the first points to consider for a microorganism in terms of its use as a probiotic. In vitro studies on the potential inflammatory or cytotoxic effects of predators with several different mammalian cell lines showed that *B. bacteriovorus* does not seem to induce a strong inflammatory response [74], with no significant alterations in cytokine levels [111,112] and no effect on cell viability [105]. In vivo experiments showed the nontoxicity of Bdellovibrios [113,114,115,116,117,118,119] on several animal models. In addition, the presence of Bdellovibrios in the human intestine makes us consider the predator to be a natural resident of the intestinal microbial ecosystem, so its functionality and stability should not be affected by the chemical–physical characteristics of the intestinal habitat. In a microbial ecosystem where different microbial species coexist in equilibrium, *B. bacteriovorus*—through the control of Gram-negative bacterial overgrowth—could preserve the coexistence of different species [121]. Further studies are required to confirm the potential to use BALOs in vivo, although available studies show that bacterial predators should be taken into account for rebuilding the gut microbial ecosystem and as an alternative to antibiotic therapies.

## Figures and Tables

**Table 1:** In vivo application of Bdellovibrio.

Bdellovibrio Strain	Prey Strain	Animal Model and Inoculation	Outcome	**References**
*B. bacteriovorus*	*Shigella flexneri*	Inoculation in eyes of rabbit model of keratoconjunctivitis	Reduced severity and no toxic effect	[118]
*B. bacteriovorus*	*Shigella flexneri*	Ileal loops of rabbits	Reduced enterosorption no toxic effect	[118]
*B. bacteriovorus MS7*	Commensal microbiota	Mice forced to drink Bdellovibrio-containing water	Predator cells were undetectable in the intestinal contents of mice	[129]
*B. bacteriovorus HD100*	*Salmonella enteritidis*	Orally administered two-day-old chicks	Reduction in the numbers of Salmonella cells in chickens cecal contents	[132]
*B. bacteriovorus 109J*	*Klebsiella pneumonia*	Respiratory and intravenous inoculations in mice	No reduction of mice viability and modest but not sustained inflammatory response	[120]
*B. bacteriovorus HD100*	*Shigella flexneri*	Zebrafish larvae	Increased survival of larvae	[123]
*B. bacteriovorus 109J*	*Klebsiella pneumonia*	Systemic infection in rats induced by tail vein inoculation	Ineffective	[121]
*B. bacteriovorus 109J*	Commensal microbiota	Intrarectal inoculation in IBD rats	No adverse effect in gut histology and inflammation	[121]
*B. bacteriovorus HD100*	*Yersinia pestis*	Mice lethal plague	Protective effect	[124]
*B. bacteriovorus HD100*	Adherent Invasive *E. coli*	*Galleria mellonella* inoculation into the *haemocoel*	Protective effect	[33]
